# A novel fluorescent sensor protein for detecting changes in airway surface liquid glucose concentration

**DOI:** 10.1042/BJ20141041

**Published:** 2014-12-01

**Authors:** Nordine Helassa, James P. Garnett, Matthew Farrant, Faaizah Khan, John C. Pickup, Klaus M. Hahn, Christopher J. MacNevin, Robert Tarran, Deborah L. Baines

**Affiliations:** * Institute of Cardiovascular and Cell Science, St George’s, University of London, London SW17 0RE, U.K; † Institute for Infection and Immunity, St George’s, University of London, London SW17 0RE, U.K; ‡ Diabetes Research Group, King’s College London, Guy’s Hospital Campus, London SE1 1UL, U.K; § Department of Pharmacology/Cell Biology & Physiology, University of North Carolina, Chapel Hill, NC, 27599 U.S.A

**Keywords:** airway epithelial cell, airway surface liquid, BADAN, glucose, glucose-binding protein

## Abstract

Both lung disease and elevation of blood glucose are associated with increased glucose concentration (from 0.4 to ~4.0 mM) in the airway surface liquid (ASL). This perturbation of ASL glucose makes the airway more susceptible to infection by respiratory pathogens. ASL is minute (~1 *μ*l/cm^2^) and the measurement of glucose concentration in the small volume ASL is extremely difficult. Therefore, we sought to develop a fluorescent biosensor with sufficient sensitivity to determine glucose concentrations in ASL *in situ*. We coupled a range of environmentally sensitive fluorophores to mutated forms of a glucose/galactose-binding protein (GBP) including H152C and H152C/A213R and determined their equilibrium binding properties. Of these, GBP H152C/A213R–BADAN (*K*_d_ 0.86 ± 0.01 mM, *F*_max_/*F*_0_ 3.6) was optimal for glucose sensing and in ASL increased fluorescence when basolateral glucose concentration was raised from 1 to 20 mM. Moreover, interpolation of the data showed that the glucose concentration in ASL was increased, with results similar to that using glucose oxidase analysis. The fluorescence of GBP H152C/A213R–BADAN in native ASL from human airway epithelial cultures *in situ* was significantly increased over time when basolateral glucose was increased from 5 to 20 mM. Overall our data indicate that this GBP is a useful tool to monitor glucose homoeostasis in the lung.

## INTRODUCTION

The respiratory tract is covered by a thin layer of fluid that lines the luminal surface of the epithelium [airway surface liquid (ASL)], which is important for lung defence against infection. In the upper airway, glucose concentrations are normally ~0.4 mM, 12.5 times lower than in plasma at ~5 mM, which serves to deprive bacteria of a potential food source and thus limits bacterial growth [1–4]. There is accumulating evidence that glucose concentrations are raised in patients (up to 4 mM) during airway inflammation (e.g. in respiratory disease), when blood glucose levels are increased (e.g. in diabetes mellitus) and, more potently, when both are present. Similar changes can also be detected in the ASL of *in vitro* models of airway epithelium in response to basolateral hyperglycaemia and pro-inflammatory stimuli [5–7]. The increase in ASL glucose concentrations makes the airways more susceptible to infection from pathogens such as methicillin-resistant *Staphylococcus aureus* and *Pseudomonas aeruginosa*.

Measurement of glucose concentrations in the airways and distal lungs of patients, in *in vivo* models of airways disease and *in vitro* models of airway epithelium is difficult because of limited accessibility, the thinness of the ASL (~7 *μ*m) and low concentrations of glucose obtained when dilution techniques are used (e.g. brochoalveolar lavage and exhaled breath condensate). Standard techniques such as glucose oxidase analysis or HPLC are concentration-limited and time-consuming respectively. Moreover, neither technique enables real-time dynamic changes in ASL glucose concentration in response to hyperglycaemic/pro-inflammatory challenge to be measured. The development of biosensors to measure glucose in the airway *in vitro* and *in vivo* will be a useful tool to better understand ASL glucose homoeostasis, how it is changed with inflammation and hyperglycaemia and how it modifies the innate immunity in the ASL.

Recently, the development of molecular glucose sensors for less invasive continuous monitoring of plasma glucose in patients has received significant interest in the field of diabetes research. One of these biosensors employs glucose/galactose-binding protein (GBP) which alters its conformation when glucose binds. This protein has been covalently linked to environmentally sensitive fluorophores which allow glucose binding to be detected as a change in fluorescence [8,9]. Native bacterial GBP has a very low dissociation constant (*K*_d_ 2 *μ*M) [8] which is not appropriate for ASL glucose measurement. However, in the present study we have used mutated forms of the GBP with altered binding constants (GBP H152C and GBP H152C/A213R), covalently bound to environmentally sensitive fluorophores. The molecular glucose sensors were characterized in terms of affinity and dynamic range, and their potential for measurement of glucose in ASL *in situ* was assessed.

## EXPERIMENTAL

### Materials

*Escherichia coli* XL10-Gold and BL21(DE3) Gold cells were purchased from Stratagene. EZ-Run protein ladder was purchased from Fisher Bioreagents and Ni^2+^-nitrilotriacetate (Ni-NTA) resin from Qiagen. IANBD, Click-iT^®^ Protein Reaction Buffer kit and iodoacetamide alkyne were obtained from Life Technologies. BADAN, oxazine derivatives (Blue Oxazine, Red Oxazine and Nile Blue) and Chromis dyes (Chromis 630 and 678) were purchased from Eurogentec, Chromeon and Cyanagen, respectively. The merocyanine dyes (Mero 53, 62 and 87) were synthesized as previously described [10–12]. Texas Red–dextran was obtained from Life Technologies.

### Expression and purification of GBP proteins

The expression vectors pET303-GBP H152C and pET303-GBP H152C/A213R were used for the production of the GBP mutants [8]. GBP proteins were overexpressed in *E. coli* BL21(DE3) Gold. Cells were grown at 37 °C and expression was induced overnight at 20 °C in the presence of 0.5 mM IPTG. Cells were pelleted by centrifugation and processed as previously described [9]. Briefly, cells were resuspended in 50 mM NaH_2_PO_4_, 300 mM NaCl, 10 mM imidazole and 5 mM tris-(2-carboxyethyl)phosphine (TCEP), pH 8, supplemented with Complete EDTA-free protease inhibitor cocktail (Roche). The suspension was incubated on ice with 1 mg·ml^−1^ lysozyme for 30 min before lysis by sonication (VibraCell, Jencons PLS), clarified by centrifugation and purified by Ni-chromatography on an ÄKTA Purifier system (GE Healthcare) at 4 °C. The 5 ml Ni-NTA column was equilibrated with 50 mM NaH_2_PO_4_, 300 mM NaCl, 10 mM imidazole and 5 mM TCEP, pH 8. The purified protein was eluted in buffer containing 300 mM imidazole and stored at −80 °C. Purity of the eluted fractions was determined by SDS/PAGE.

### Measurement of protein concentration

Protein concentration was determined using a Nanodrop 1000 spectrophotometer (Thermo Scientific) with a molar absorption coefficient (*ε*_o_) of 37,930 M^−1^·cm^−1^, calculated from the amino acid composition [13].

### Protein labelling using thiol-reactive dyes (IANBD, BADAN, mero53, mero62 and mero87)

For labelling with UV-excitable fluorophores, GBP proteins (100 *μ*M) were incubated in the presence of 10-fold excess of dye (IANBD or BADAN) overnight at 4 °C in PBS and 5 mM TCEP, pH 7.4 [14]. The excess of dye was removed by gel filtration using a PD-10 column (GE Healthcare). For labelling with merocyanine dyes, 100 *μ*M of protein were mixed with 5-fold excess of dye for 2 h at room temperature in PBS, pH 7.4. The excess of dye was removed by extensive dialysis in PBS, pH 7.4 at 4 °C (Slide-a-Lyser 10 kDa cut-off, Pierce).

### Protein labelling using click chemistry for oxazine derivatives (Blue Oxazine, Red Oxazine, Nile Blue) and chromis dyes (Chromis 630, Chromis 678)

First, GBP-alkyne was obtained by incubating 100 *μ*M GBP with 10-fold excess of iodoacetamide alkyne in PBS and 2.5 mM TCEP, pH 7.4, for 2 h at room temperature. The excess of iodoacetamide alkyne was removed by extensive dialysis in PBS, pH 7.4, at 4 °C (Slide-a-Lyser 10 kDa cut-off, Pierce). The second step of the process consisted of labelling the GBP-alkyne with the dye azide using the Click-iT^®^ Protein Reaction Buffer kit following the manufacturer’s recommendations. Briefly, 40 *μ*M GBP-alkyne was incubated with 2.5-fold excess of dye in 100 *μ*M reaction buffer containing 25 *μ*l of copper sulfate reagent and 25*μ*l of kit additive 1, for 1 h at room temperature (*V*_t_ = 500 *μ*l). The excess of dye was removed by extensive dialysis in PBS, pH 7.4, at 4 °C (Slide-a-Lyser 10 kDa cut-off, Pierce).

### Equilibrium glucose binding

Equilibrium glucose-binding curves were obtained by continuous titration followed by corrections for dilution and photobleaching. To determine the fluorescence dynamic range (*F*_max_/*F*_0_), the co-operativity (*n*) and the dissociation constant (*K*_d_) of the labelled GBP proteins, glucose affinity assays were performed using an automated syringe pump (ALADDIN 1000, WPI). Labelled GBP proteins (40–100 nM) were titrated continuously with D-glucose at 10–20 *μ*l/min flow rate in a stirred 3 ml cuvette, in PBS, pH 7.4. Fluorescence was measured at fluorophore excitation and emission wavelength peaks (Table 1) at 20 °C on a Fluorolog3 spectrofluorimeter (Horiba Scientific). Data are expressed as the means ± S.E.M. of triplicates. The fluorescence changes were normalized and fitted to the Hill equation $Y=B_{\max}\times X^{n}/({K_{d}}^{n}+X^{n})$ using Prism GraphPad 6 software.

Fluorescence stability at 37 °C of GBP H152C/A213R–BADAN (100 nM) was monitored over 7 h (*λ*_exc_ 387 nm and *λ*_em_ 535 nm) under physiological ionic strength (PBS, pH 7.4).

### Cell culture and ASL glucose measurement

#### Calu-3 cells

The human adenocarcinoma-derived cell line, Calu-3 [15], was grown in Eagle’s minimal essential medium (EMEM) plus 10 %FBS, 2 mM L-glutamine, 100 units/ml penicillin, 100 *μ*g/ml streptomycin, and 1 % non-essential amino acids (Sigma) and incubated in humidified air containing 5 % CO_2_ at 37 °C. Calu-3 cells were seeded on to clear Costar Transwell^®^ inserts (0.45-*μ*m pore size) at 250 000 cells/cm^2^ to form confluent polarized monolayers, as previously described [16]. Experiments were carried out 10–14 days post-seeding. Calu-3 cells were bathed with 100 *μ*l of glucose-free physiological salt solution (PSS) (117 mM NaCl, 25 mM NaHCO_3_, 4.7 mM KCl, 1.2 mM MgSO_4_, 1.2 mM KH_2_PO_4_ and 2.5 mM CaCl_2_) on the apical surface and 1 ml of PSS on the basolateral side supplemented with 1, 2, 5, 10, 15 or 20 mM glucose, in a 95 % O_2_/5 % CO_2_ gassed incubator at 37 °C. The ASL was collected after 1 h or 24 h. GBP–BADAN (2 *μ*l) was added to 48 *μ*l of ASL and fluorescence measured on a Fluorolog3 spectrofluorimeter (Horiba Scientific) at *λ*_exc_ 387 nm and *λ*_em_ 535 nm. The remaining 50 *μ*l of ASL was analysed using glucose oxidase (Glucox).

#### Primary human bronchial epithelial cells

Primary human bronchial epithelial cells (HBECs) were obtained from endobronchial brushings or extracted from explanted lungs and cultured as previously described [17–19]. HBECs were obtained in accordance with approval from the relevant ethics committees: The University of North Carolina at Chapel Hill Biomedical Institutional Review Board (protocol #03-1396). Cells were transferred on to transwell permeable supports and grown at air–liquid interface to form confluent monolayers. Cells were studied 3–5 weeks post-seeding. For ASL glucose studies, cells were transferred into Hepes-buffered PSS (24 mM Hepes, 101 mM NaCl, 12 mM NaHCO_3_, 1.2 mM MgCl_2_, 1.2 mM CaCl_2_·2H_2_O and 5.2 mM KCl) and the apical surface washed with 100 *μ*l. PSS (20 *μ*l) containing 0.1 mg/ml Texas Red–dextran 10 000 *M*_r_ (Life Technologies) and 2 *μ*l of a 50 *μ*M solution of GBP–BADAN were added to the apical surface and the fluorescence emission of Texas Red and BADAN was analysed from five different areas of the transwell surface using a Infinite M1000 plate reader (Tecan). To initiate the experiment, 5 mM D-glucose, 10 mM D-glucose, 20 mM D-glucose, 5 mM D-glucose plus 5 mM L-glucose (osmotic control for 10 mM D-glucose), or 5 mM D-glucose plus 15 mM L-glucose (osmotic control for 20 mM D-glucose), was added to the basolateral chamber and fluorescent readings of the apical surface taken hourly. Data were analysed as the ratio of GBP–BADAN/Texas Red–dextran fluorescence (to correct for changes in ASL fluid volume).

#### Statistical methods

Groups were compared using ANOVA with Tukey’s post-hoc test. Results are presented as means ± S.E.M., unless otherwise stated.

## RESULTS

### Equilibrium glucose binding to GBP H152C and H152C/A213R

GBP proteins were highly purified on a single-step affinity chromatography column (Figure S1), allowing accurate concentration measurements by spectrophotometry. One litre of culture yielded in 200–250 mg of purified protein.

**Figure 1:**
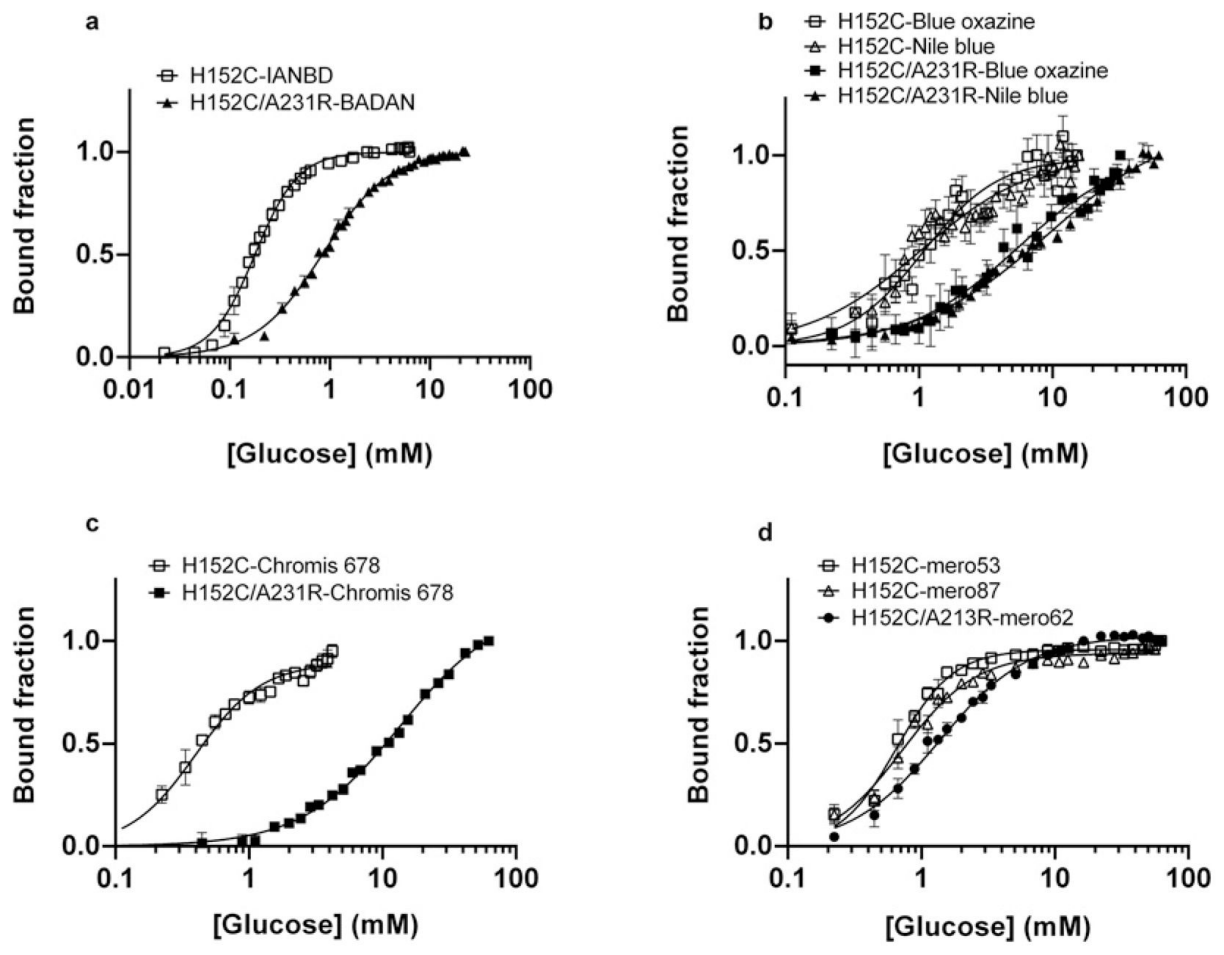
Equilibrium glucose titrations for fluorescently labelled GBP H152C and GBP H152C/A213R Equilibrium glucose titrations for GBP H152C and GBP H152C/A213R, labelled with UV-excitable dyes (**A**), oxazine derivatives (**B**), Chromis 678 (**C**) and Merocyanine dyes (**D**). Titrations were carried out in at least triplicates and the curves represent the means ± S.E.M. Data were fitted to the Hill equation using GraphPad Prism 6.0 and the fitted parameters are shown in Table 1.

Glucose equilibrium binding for labelled GBP H152C and H152C/A213R showed that using UV-excitable environmentally sensitive dyes such as BADAN and IANBD offered the highest fluorescence dynamic range (*F*_max_/*F*_0_) ranging from 2.7 to 3.6 (Table 1). GBP labelled with long-wavelength excitable dyes showed a maximum fluorescence change upon glucose binding of 80 %for GBP H152C coupled to merocyanines. The dissociation constant (*K*_d_) for GBP H152C and GBP H152C/A213R ranged from 0.046 ± 0.002 mM to 0.68 ± 0.02 mM and from 0.86 ± 0.01 mM to 15.2 ± 0.3 mM, respectively (Figure 1 and Table 1). For most labelled glucose-binding proteins, glucose binding did not occur with co-operativity as determined by analysis of the Hill coefficient (*n*). All the parameters obtained from the glucose titration curves are summarized in Table 1. GBP H152C and GBP H152C/A213R labelled with Red Oxazine and Chromis 630 did not show a significant fluorescence change upon glucose binding (results not shown).

### Specificity of GBP H152C/A213R–BADAN (GBP–BADAN) for D-glucose

**Figure 2:**
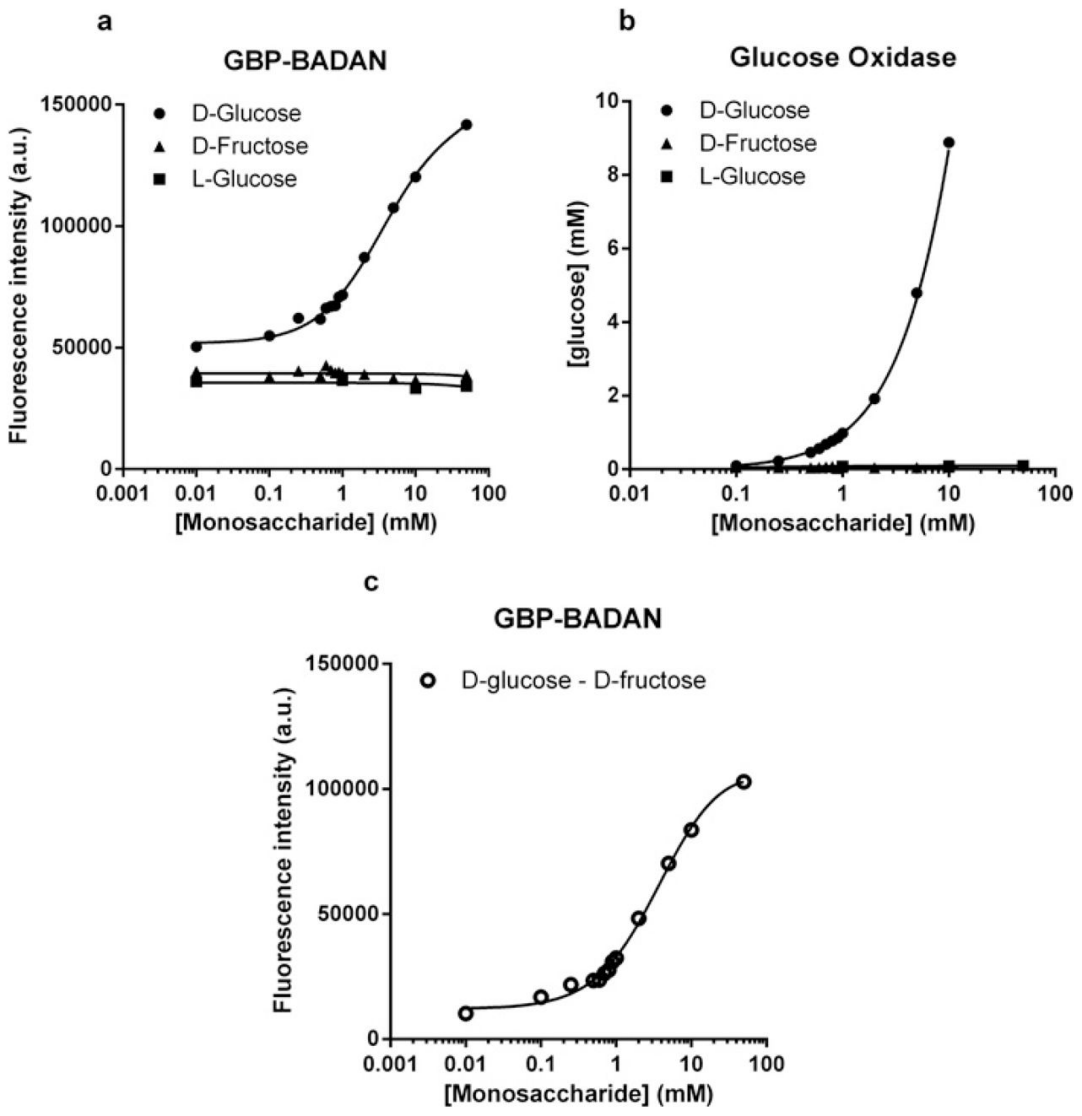
Titration of BADAN-labelled GBP H152C/A213R (GBP–BADAN) with monosaccharides Titration of BADAN-labelled GBP H152C/A213R (GBP–BADAN) (**A**) with D-glucose, L-glucose or D-fructose, in PSS compared with glucose oxidase analysis (**B**). Fluorescence intensity titration curve for GBP–BADAN (D-glucose minus D-fructose) (**C**). This curve was used to interpolate data shown in Figure 3. Titrations were carried out in triplicate and the curves represent the means ± S.E.M. Data were fitted to the Hill equation using GraphPad Prism 6.0. a.u., arbitrary unit (s).

The specificity of glucose induced fluorescence of GBP–BADAN was analysed in PSS by comparing fluorescence in the presence of D-glucose, its isomer L-glucose and D-fructose. Fluorescence did not change with increasing D-fructose or L-glucose concentration but exhibited a similar dose response to D-glucose as described above (Figure 2A). There was a significant difference in the mean values between 0.01 and 0 mM D-glucose (i.e. in the presence of L-glucose) (*P* = 4.6944×10^−5^, *n* = 3). This indicated that the limit of detection was below 0.01 mM. However, we did not explore this further because values below 0.01 mM were not relevant to our study system. This was also the case for glucose concentrations above 50 mM. The calculated value for precision (3.3×S.D./slope for the linear part of the graph shown in Figure 1) was 0.27 mM.

### Comparison of GBP–BADAN fluorescence with glucose oxidase measurement of glucose concentration

Glucose oxidase analysis showed similar D-glucose specificity, with mM values obtained for increasing concentrations of L-glucose and D-fructose being unchanged from 0 (Figure 2B). Interestingly, GBP–BADAN exhibited changes in fluorescence at low concentrations of D-glucose (from 0.01 mM; Figure 2A). Changes in low glucose concentrations were less discernable by glucose oxidase analysis. Furthermore, changes in fluorescence could be detected at concentrations up to 50 mM where no accurate and repeatable readings could be determined at this concentration by glucose oxidase analysis calibrated for standard glucose measurement (glucose oxidase data not shown). Therefore, GBP–BADAN appears to be a more sensitive probe than glucose oxidase and can measure glucose over a broader range of concentration.

### GBP–BADAN to measure hyperglycaemia-induced changes in Calu-3 ASL glucose concentration

Serous and mucous cells of the airway submucosal glands make a significant contribution to the volume, composition and consistency of the ASL *in vivo*. Calu-3 cells, a heterogenous cell line derived from human submucosal glands, secrete a protein-rich fluid containing mucins, antimicrobials and enzymes [15,20,21]. Calu-3 monolayers were used to determine whether the protein-rich ASL would affect the stability of GBP–BADAN to accurately measure glucose content. Fluid was added to the apical surface and removed after 1 and 24 h, and glucose concentrations measured by glucose oxidase and GBP–BADAN fluorescence. Hyperglycaemia was mimicked by increasing the D-glucose concentration of the basolateral fluid (from 1 to 20 mM), as previously described [6].

**Figure 3:**
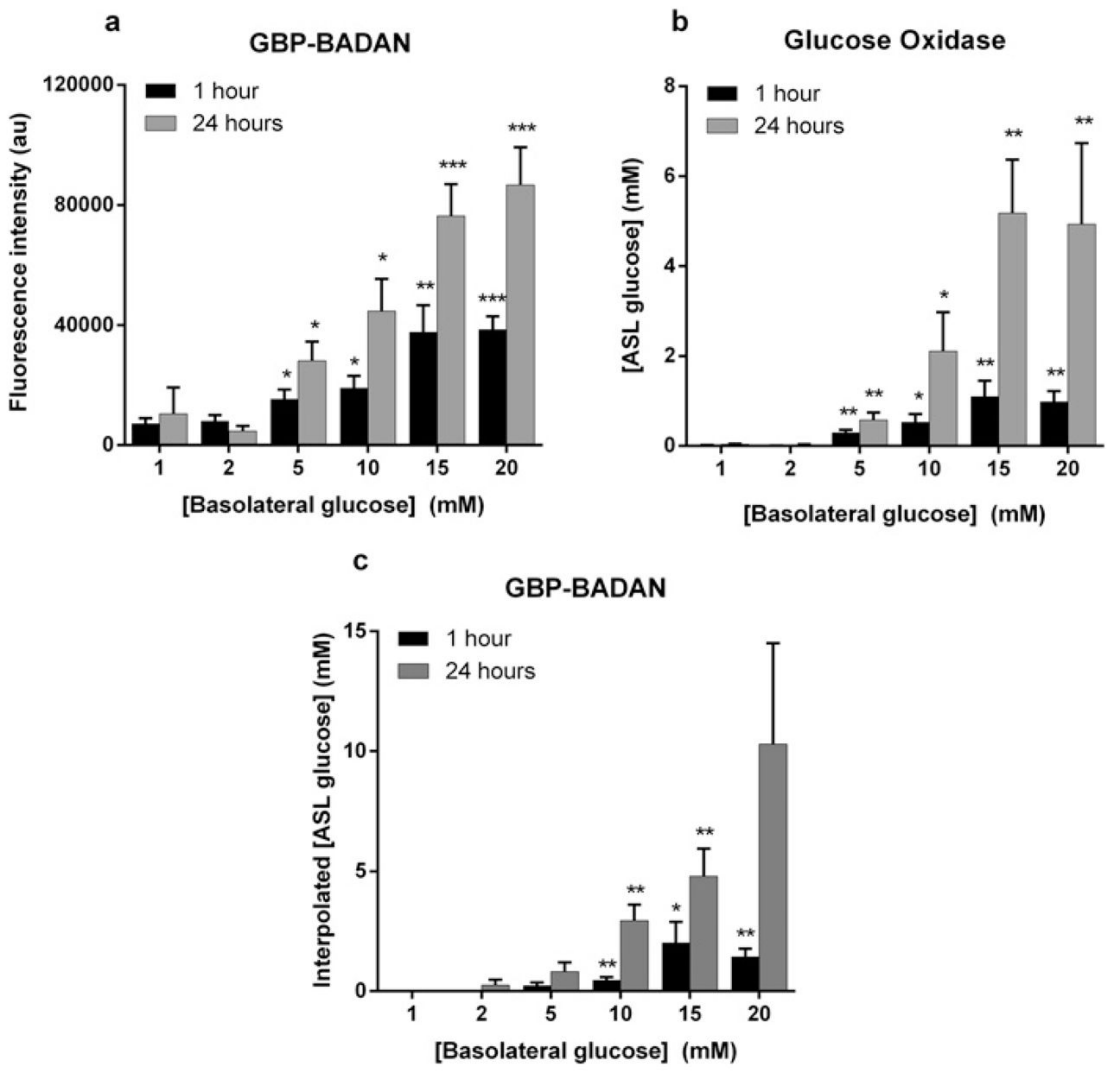
Determination of glucose concentrations in ASL Determination of glucose concentrations in ASL removed from Calu-3 monolayers grown at air–liquid interface in different basolateral glucose concentrations for 1 and 24 h measured by fluorescence (**A**) and glucose oxidase analysis (**B**), using BADAN-labelled GBP H152C/A213R (GBP–BADAN). Glucose concentration calculated by interpolation of fluorescence intensity of GBP–BADAN (**C**), using graph shown in Figure 2(c). Data represent the means ± S.E.M. Significantly different from 1 mM glucose: *P < 0.05, **P < 0.01, ***P < 0.001, *n* = 4.

GBP–BADAN fluorescence in ASL increased significantly when basolateral D-glucose was elevated from 1 to 5 mM and above (*P* < 0.05, *n* = 4) with maximal values occurring when basolateral glucose was 15–20 mM (*P* < 0.01, *n* = 4). ASL glucose concentrations were higher in samples obtained after 24 h compared with 1 h after elevation of basolateral D-glucose (Figure 3A).

A similar pattern was observed when glucose concentration in the samples of ASL was analysed by glucose oxidase method. At 1 h, glucose concentration in ASL was very low (0.007 ± 0.002 mM) and at the limit of detection when basolateral D-glucose was 1–2 mM but significantly increased when basolateral glucose was raised to 5 mM (0.3 ± 0.06 mM in ASL; *P* < 0.01, *n* = 4) and was highest when basolateral D-glucose was increased to 15 mM (1.1 ± 0.3 mM; *P* < 0.01, *n* = 4). ASL glucose further increased after 24 h rising to 0.6 ± 0.17 and 5.0 ± 1.2 mM when basolateral D-glucose was 5 and 15 mM, respectively (Figure 3B). Importantly, ASL glucose concentration remained lower than basolateral glucose at any concentration tested.

Airway epithelial monolayers maintained their transepithelial resistance when glucose in the basolateral chamber was replaced with fructose, as previously reported [22]. As fructose did not bind to GBP–BADAN, ASL from cells grown in basolateral fructose was used to obtain ASL in which glucose was not present and correct for background fluorescence. Then, using the standard curve (Figure 2C), ASL glucose concentration can be determined. Importantly, the interpolated ASL glucose concentrations were similar to those determined by glucose oxidase analysis at 0.2 ± 0.1 and 0.8 ± 0.4 mM when basolateral D-glucose was 5 mM and 2.0 ± 0.8 and 4.8 ± 1.1 mM when basolateral D-glucose was 15 mM at 1 and 24 h respectively (Figure 3C). These data indicate that GBP–BADAN fluorescence can be used to accurately measure ASL glucose at similar sensitivity to glucose oxidase analysis.

### GBP–BADAN to measure dynamic changes in HBEC ASL glucose concentration in response to hyperglycaemic challenge *in situ*

**Figure 4:**
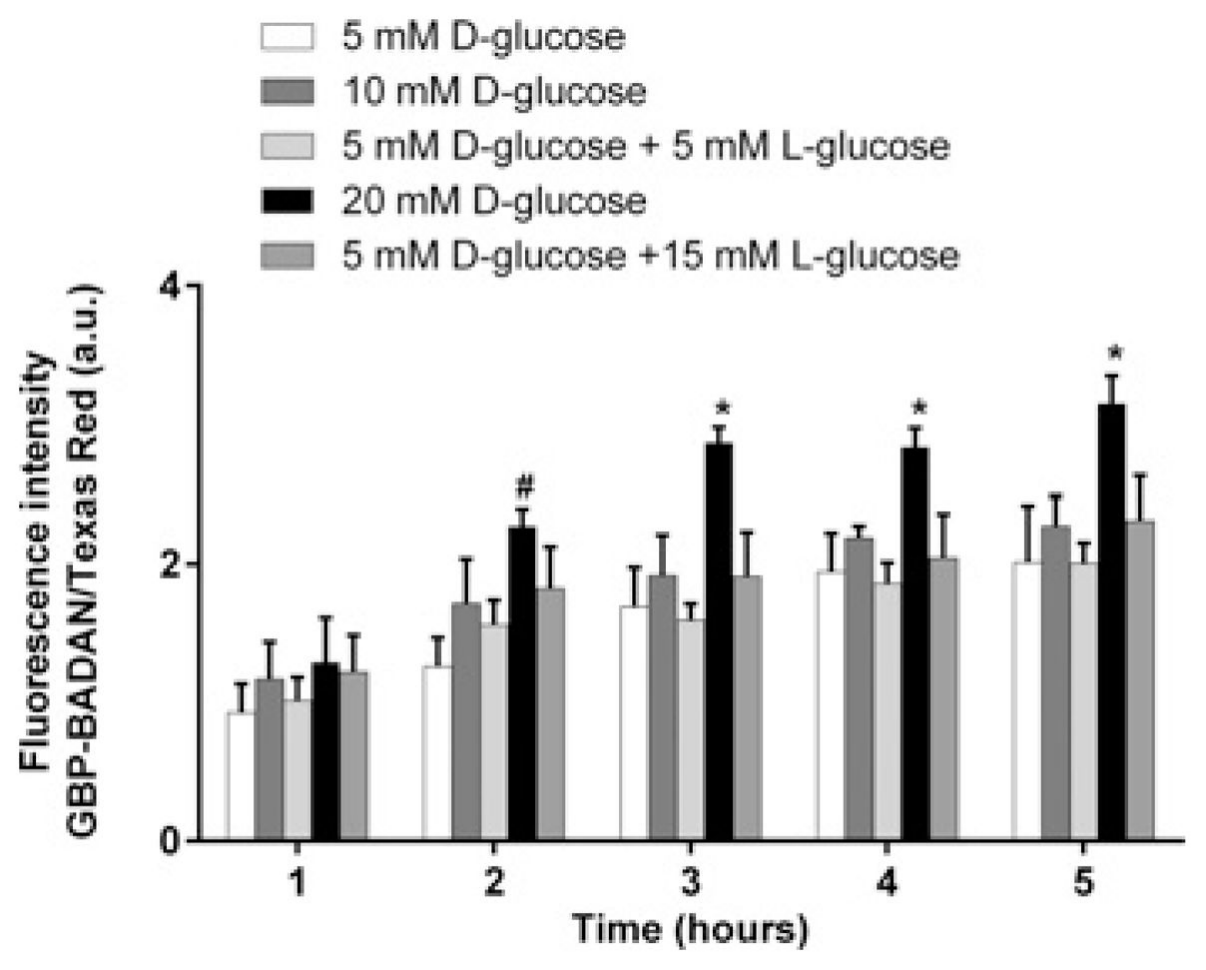
Dynamic changes in primary human bronchial ASL glucose concentration *in situ* Dynamic changes in primary human bronchial epithelial ASL glucose concentration *in situ*, measured as fluorescence of BADAN-labelled GBP H152C/A213R in response to raised basolateral glucose over time. Data are shown as means ± S.E.M. Significantly different from all other columns at time point: *P < 0.05; significantly different from 5 mM D-glucose only: #*P* < 0.05, *n* = 4.

Having shown that GBP–BADAN could be used to measure changes in ASL glucose concentrations collected from Calu-3 monolayers, we investigated whether the GBP assay could be used to detect dynamic changes *in situ,* in response to elevated basolateral glucose, by applying GBP–BADAN directly to the apical surface of primary HBECs. Applying even small volumes of fluid to the apical surface of HBECs induces fluid absorption [23]. Therefore, to control for the effect of ASL volume changes on glucose concentration, we used Texas Red-labelled dextran together with GBP–BADAN and calculated the change in fluorescence of GBP–BADAN/Texas Red ratio. Using this methodology, we found that fluorescence increased in the ASL of all HBECs after 1 h and reached peak fluorescence after 3 h (Figure 4). Although no significant difference in GBP–BADAN fluorescence was observed in the ASL of HBECs bathed in 10 mM basolateral glucose compared with 5 mM control, increasing basolateral glucose to 20 mM significantly enhanced fluorescence after 2 h (*P* < 0.05, *n* = 4). The increase in GBP–BADAN fluorescence was maintained throughout the 5 h time-course and was also significantly greater than cells bathed in 5 mM D-glucose plus 15 mM L-glucose (from 3 h onwards; *P* < 0.05, *n* = 4), consistent with a glucose-induced change in fluorescence rather than one induced by a difference in osmotic gradients.

The fluorescence of GBP–BADAN was monitored over 7 h at 37 °C and no significant change in fluorescence was observed indicating that the labelled protein was stable over this time course (Figure S2).

## DISCUSSION

The world burden of diabetes is increasing both as an independent disease and as co-morbidity with other chronic conditions. In the lung, evidence is accumulating that hyperglycaemia associated with Type 1 and Type 2 diabetes results in increased glucose concentration in the ASL which promotes infection, particularly when associated with underlying respiratory disease [1,5,6,24–27]. However, measurement of real-time changes of ASL glucose *in vitro* and *in vivo* has not previously been possible.

Evidence indicates that glucose concentrations in the ASL range from approximately 0.4 to 4.0 mM *in vivo* and *in vitro* [1,6,7,24]. In order to efficiently monitor glucose concentrations in ASL *in vivo*, the biosensors should have a *K*_d_ between 0.1 and 10 mM and a high fluorescence dynamic range upon glucose binding. We initially disregarded GBP H152C (which contains a mutation to enable covalent labelling of fluorophores via the cysteine amino acid) because when it was bound to BADAN the *K*_d_ was 2 *μ*M, somewhat below our ideal range [8,28]. However, our data showed that when bound to other fluorophores, particularly IANBD, a shift in the *K*_d_ to higher, more useful, values in the millimolar range could be achieved. Similar increases in *K*_d_ were also observed when these fluorophores were attached to GBP H152C/A213R. These data indicate that the molecular structure of the fluorophore bound to these proteins can significantly modify affinity for the ligand, consistent with the findings of others [9,29]. Unfortunately, linking the double mutant to IANBD increased the *K*_d_ to ~32 mM (results not shown), making it unlikely to be suitable for ASL glucose measurement, although it gave the next best dynamic range after BADAN. Interestingly, all the longer wavelength fluorophores, which were designed for *in vivo* imaging, were less sensitive to environmental changes and had lower fluorescence dynamic ranges. These fluorophores were based on optimization of both environmental sensitivity and brightness, as these factors together determine sensitivity in living cells [30]. Of these dyes, GBP H152C/A213R–mero62 was the most promising for further development with a *K*_d_ of 1.4 mM and a fluorescence dynamic range of 1.7.

GBP H152C/A213R–BADAN (GBP–BADAN) gave the most useful *K*_d_ (0.86 ± 0.01 mM) and the largest fluorescence dynamic range (*F*_max_/*F*_0_ 3.5) and exhibited clear specificity for D-glucose. Moreover, we found that it was a better sensor over the range of glucose concentrations required than using the glucose oxidase method. This was in part due to technical limitations of the sensing machine. That is, in order to obtain readings at concentrations below 1 mM, the Glucox analyser has to be recalibrated differently from settings used to obtain blood glucose readings (2–20 mM). This meant that glucose concentrations in the higher range *>*10 mM did not give a consistent output value. When measuring glucose in samples of ASL removed from the surface of cells, GBP–BADAN and glucose oxidase analysis showed similar changes with increasing basolateral glucose. Glucose oxidase analysis gave absolute glucose concentrations whereas we obtained fluorescence intensity for GBP–BADAN. We utilized the finding that the presence of basolateral D-fructose (which maintains cell survival) is not sensed by GBP–BADAN to obtain background fluorescence readings for ASL with no glucose. This enabled us to convert the mean fluorescence data into glucose concentration (mM) by interpolation of the standard curve. Glucose concentration values obtained by fluorescence and the glucose oxidase data were similar, providing support for the use of GBP–BADAN to quantify glucose in ASL. Most importantly, the data we gained from these experiments showed that glucose concentration in ASL increased with raised basolateral glucose but concentrations remained significantly lower in ASL, consistent with our previous findings *in vitro* and *in vivo* [6,7,24].

Work by others has shown that highly technical glucose sensor technology using ZnO-coated microelectrodes or a phosphorescence sensing material [crystalline iridium(III)-Zn(II) co-ordination polymers], which utilize glucose oxidase production of H_2_O_2_ to quantify glucose, can be useful in measuring glucose in small fluid volumes such as ASL and over similar ranges of glucose (0.1–6 mM) [26,31,32]. However, the benefit of a fluorescent molecular glucose sensor that is easy to produce and can be used to rapidly measure real-time (and reversible) changes in glucose concentration in ASL overlying cells *in situ* is particularly attractive. In a unique experiment, we used GBP–BADAN applied directly to the ASL of HBECs *in situ* and showed that glucose concentration rises faster and to higher levels over a time course of 5 h when basolateral glucose concentration was increased from 5 to 20 mM. These data support our model of glucose homoeostasis where raised basolateral glucose concentration increases the driving force for glucose diffusion into ASL. The resultant ASL glucose concentration is the net balance between diffusion and glucose uptake by the epithelial cells. Thus, if uptake remains the same, increased diffusion would result in elevated ASL glucose [33,34].

These experiments now open the way for development of fluorophore-labelled GBPs as biosensors, for use *in vitro* and *in vivo*, to further understand how hyperglycaemia and respiratory disease change ASL glucose concentration in the proximal and distal lung. We now intend to use GBP–BADAN applied directly to ASL of proximal and distal airway cells *in vitro* to explore factors regulating glucose homoeostasis. *In vivo*, we have previously used glucose oxidase sticks to detect glucose in human nasal secretions. We have shown that changes in glucose concentration in nasal secretions are representative of the airway as a whole [24,35,36]. However, these sticks are no longer available, colour change interpretation was subjective and dynamic changes were difficult to measure. Therefore, immobilization of GBP–BADAN and its incorporation into a small fibre-optic probe (<1 mm diameter) for insertion into the nasal cavity, could prove a more accurate and minimally invasive biosensor for detection of glucose in nasal secretions [37]. During our *in vitro* experiments we did not observe any changes in transepithelial resistance which indicated GBP–BADAN toxicity, but this will need to be tested further. Nevertheless, we hope such tools will be helpful to find new ways to reduce glucose in ASL and help combat respiratory infections.

## Supplementary Material

supplemental

## Figures and Tables

**Table 1:** Summary of the biophysical characteristics of GBP-labelled mutants

Protein	Fluorophore	*λ*_exc_/*λ*_em_	*K*_d_ (mM)	*n*	*R*^2^	*F*_max_/*F*_0_
GBP H152C	IANBD	470/535	0.18 ± 0.01	1.8 ± 0.1	0.98	3.6
	Blue Oxazine	637/666	0.22 ± 0.03	0.7 ± 0.1	0.57	1.3
	Nile Blue	625/670	0.19 ± 0.02	0.8 ± 0.1	0.51	1.4
	Chromis 678	677/701	0.046 ± 0.002	1.4 ± 0.1	0.93	1.4
	mero53	594/618	0.65 ± 0.01	1.8 ± 0.1	0.93	1.8*
	mero87	591/616	0.68 ± 0.02	1.1 ± 0.1	0.90	1.8*
GBP H152C/A213R	BADAN	387/535	0.86 ± 0.01	1.2 ± 0.1	0.99	3.5
	Blue Oxazine	637/666	4.6 ± 0.3	1.1 ± 0.1	0.80	1.3
	Nile Blue	625/670	11.4 ± 0.5	0.8 ± 0.1	0.93	1.7
	Chromis 678	677/701	15.2 ± 0.3	1.1 ± 0.1	0.98	1.4
	mero62	586/608	1.4 ± 0.1	1.2 ± 0.1	0.97	1.7

The asterisks (*) indicate that fluorescence was decreased upon glucose binding.

## Abbreviations

ASL: airway surface liquid
EMEM: Eagle’s minimal essential medium
GBP: glucose/galactose-binding protein
HBEC: human bronchial epithelial cell
Ni-NTA: Ni^2+^-nitrilotriacetate
PSS: physiological salt solution
TCEP: tris-(2-carboxyethyl)phosphine
