# Supplementary material for: A novel fluorescent sensor protein for detecting changes in airway surface liquid glucose concentration
Source: Biochem J. Author manuscript; Available in PMC 2015 Mar 12. (PMC4357280; doi:10.1042/BJ20141041)
Supplement: supplemental [file NIHMS664088-supplement-supplemental.pdf]

Supplemental Figures

Figure S1

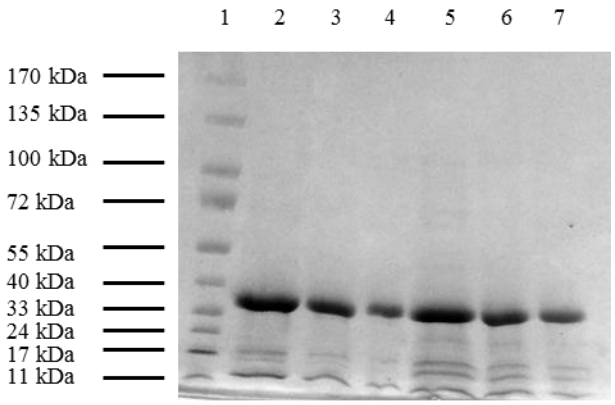

Figure S2

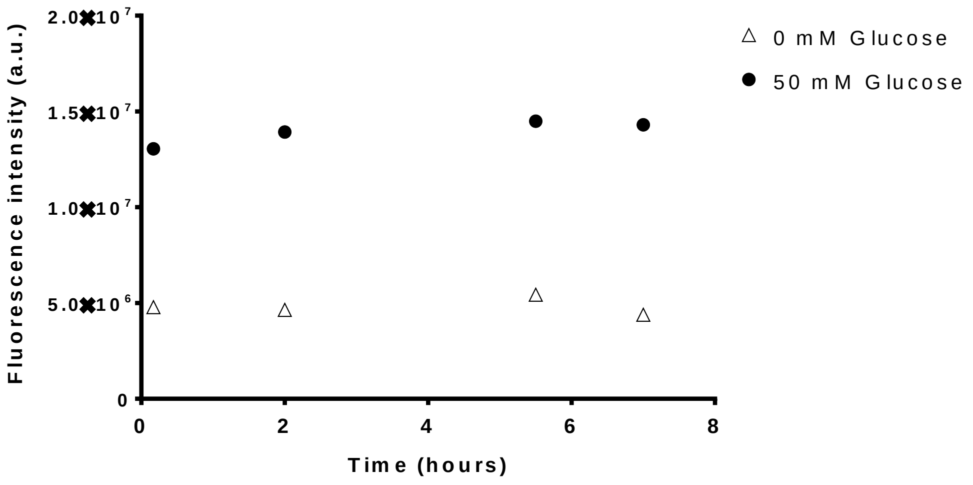
0 mM Glucose
50 mM Glucose
Fluorescence intensity (a.u.)
2.0×10⁷
1.5×10⁷
1.0×10⁷
5.0×10⁶
0
0
2
4
6
8
Time (hours)

Supplemental Figure legends

Figure S1. Purity of GBP H152C and H152C A213R. Coomassie Brilliant Blue-stained gradient SDS-PAGE analysis of GBP, following Ni-NTA chromatography. Lane 1: EZ-Run protein ladder, Lane 2-4: 10 μg, 5 μg and 2.5 μg of purified GBP H152C, Lane 5-7: 10 μg, 5 μg and 2.5 μg of purified GBP H152C A213R.

Figure S2. Fluorescence stability of labelled GBP at 37 ^{o}C. Stability of GBP H152C A213R-BADAN was monitored in the (△) absence and (●) 50 mM glucose after 10 min, 2 h, 5.5 h and 7 h at 37 ^{o}C. Fluorescence was measured at 387 nm excitation and 535 nm emission.
